# Catastrophic stroke in a patient with left ventricular non-compaction

**DOI:** 10.1530/ERP-18-0015

**Published:** 2018-06-19

**Authors:** Sothinathan Gurunathan, Roxy Senior

**Affiliations:** 1 Department of Cardiology, Northwick Park Hospital, Harrow, UK; 2 Department of Cardiology, Royal Brompton Hospital, National Heart and Lung Institute, Imperial College, London, UK

**Keywords:** 2D echocardiography, noncompaction, magnetic resonance imaging

## Abstract

We present the case of a 32-year-old man who presented with a remote history of chest pain and was diagnosed with non-compaction cardiomyopathy on echocardiography. On presentation, he was relatively asymptomatic with normal cardiac function. Unfortunately, he presented 1 year later with a catastrophic embolic stroke.

**Learning points:**

- Left ventricular non-compaction (LVNC) is a myocardial disorder characterised by prominent left ventricular (LV) trabeculae, a thin compacted layer and deep intertrabecular recesses.
- Two-dimensional echocardiography with colour Doppler is the study of choice for diagnosis and follow-up of LVNC. CMR serves an important role where adequate echocardiographic imaging cannot be obtained.
- LVNC is associated with high rates of mortality and morbidity in adults, including heart failure, thromboembolic events and tachyarrhythmias.

## Background

Left ventricular non-compaction (LVNC) is a rare cardiomyopathy and should be considered in the differential diagnosis of patients presenting with unexplained heart failure. In most cases, echocardiography in experienced hands is sufficient to make the diagnosis. Major complications are common, as this case highlights, even in patients where cardiac function is normal. Prevention of embolic complications is an important aspect of treatment in LVNC, and long-term anticoagulation should be considered.

## Case presentation

A 32-year-old gentleman presented to our institution with non-specific chest pains. He was well otherwise, with no past medical history of note. His maternal uncle had been diagnosed with hypertrophic cardiomyopathy. His presenting ECG was normal.

## Investigation

**Figure 1:**
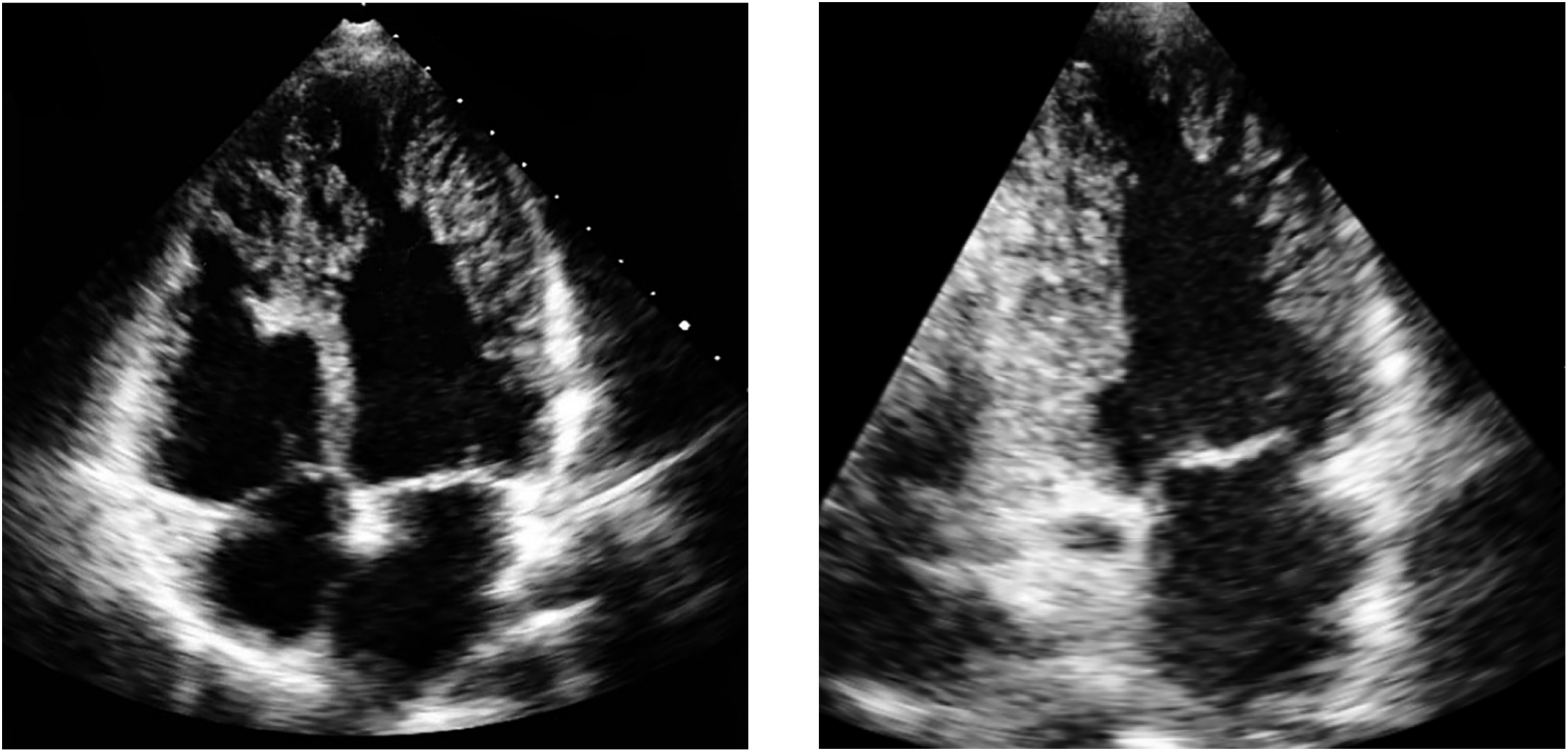
Apical four-chamber and two-chamber views on echocardiography demonstrating prominent trabeculations in both ventricles with deep intertrabecular recesses.

**Figure 2:**
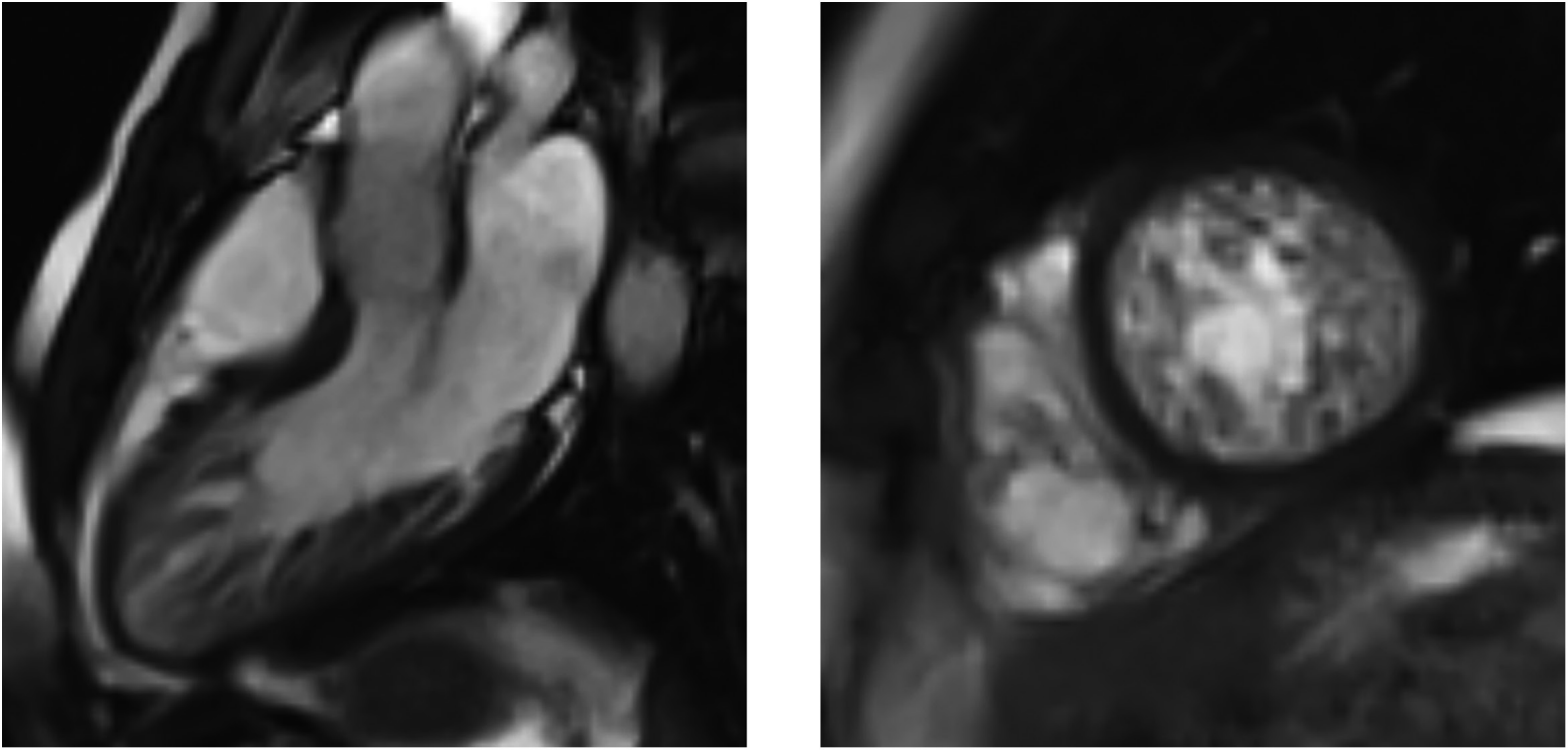
CMR confirms the diagnosis of biventricular non-compaction.

Transthoracic echocardiography demonstrated findings of LVNC with extensive prominent trabeculations in both ventricles and deep intertrabecular recesses (Fig. 1). Despite extensive biventricular myocardial involvement, there was normal systolic function and diastolic function. Cardiac magnetic resonance (CMR) confirmed LVNC, with extensive involvement of the left ventricle and sparing of the basal inter-ventricular septum (Fig. 2). The right ventricle was heavily trabeculated consistent with the echocardiographic findings. At the left ventricular free wall, mid-ventricular level, the ratio of depth of non-compacted to compacted myocardium was 2.6:1. There was no evidence of myocardial fibrosis. CMR confirmed normal body surface area indexed volumes and systolic function. To exclude arrhythmias, he underwent 48-h ECG monitoring, which was normal. He was kept under follow–up, and his two children were to be screened in due course.

## Treatment and outcome

**Figure 3:**
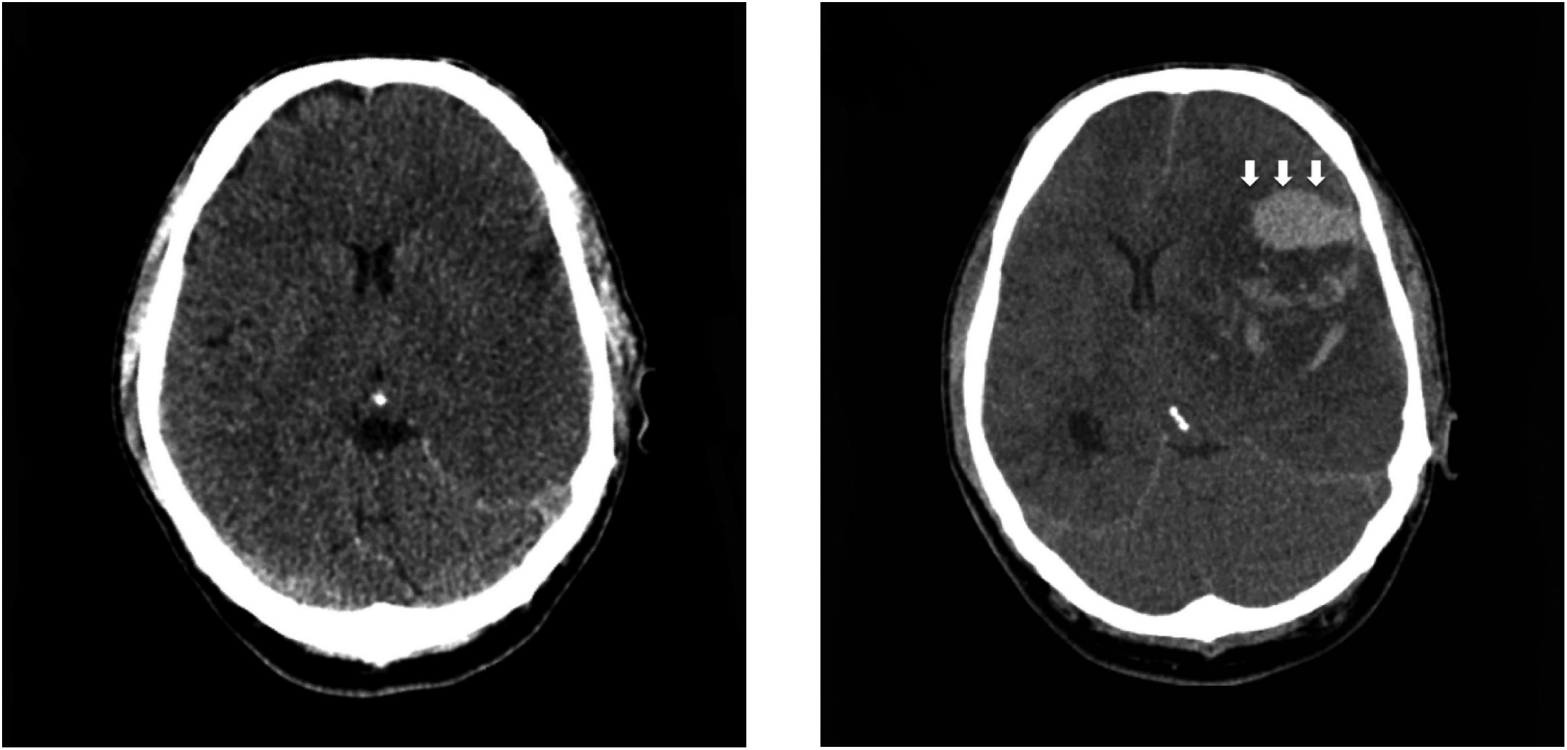
Initial CT head shows extensive loss of grey-white matter differentiation and sulcal effacement affecting the left cerebral hemisphere, consistent with large MCA infarction. Further CT head following neurological deterioration demonstrates haemorrhagic transformation within the infarct (white arrows) with mass effect.

He presented over 1 year later with collapse and reduced conscious level. A CT head demonstrated extensive left middle cerebral artery (MCA) territory infarction. On CT intracranial angiography, there was thrombus occupying the M1 and M2 branches of the left MCA. There was normal opacification of the carotid arteries and the vertebrobasilar system with no evidence of dissection flap or stenosis. In addition, his thrombophilia screen and bubble study were negative, and there was no history of illicit drug use. A further CT head 2 days later, following neurological deterioration, demonstrated haemorrhagic transformation within the infarct with associated mass effect (Fig. 3), requiring decompressive craniotomy for elevated intracranial pressure. Since other causes of thromboembolic stroke had been excluded, he was commenced on warfarin for probable thrombus developing within the non-compacted layer, causing the stroke.

## Discussion

LVNC is a myocardial disorder characterised by prominent trabeculae, inter-trabecular recesses and a left ventricular myocardium with two distinct layers: compacted and non-compacted. Although the classical clinical presentation is with heart failure, arrhythmias and embolic events, presentation is highly variable ranging from an incidental finding in an asymptomatic patient to end-stage heart failure. Biventricular involvement is a rare entity, the prognostic implications of which are unknown. Two-dimensional echocardiography with colour Doppler is the study of choice for diagnosis and follow-up of LVNC. Colour Doppler or contrast echocardiography show evidence of deep, perfused intertrabecular recesses. CMR serves a particularly important role for patients in whom adequate echocardiographic imaging cannot be obtained (1, 2).

In LVNC, mural thrombi may develop within the inter-trabecular recesses where blood flow is sluggish, which can cause systemic embolisation. *In vivo* blood clots within the non-compacted layer have been documented in patients with embolic events (3). During long-term follow-up, thromboembolic events occurred in 24% of patients in one series (4). Thus, prevention of embolic complications is an important aspect of treatment in LVNC. This is particularly true for patients with left ventricular dysfunction (fractional shortening <25% or EF <40%), atrial fibrillation, a history of thromboembolic complications, known ventricular thrombi, chamber dilatation and evidence of spontaneous contrast (5). Some investigators recommend long-term prophylactic anticoagulation for all patients with LVNC regardless of whether they have experienced thromboembolic complications and irrespective of the degree of left ventricular dysfunction. Since there is no robust data to support either approach, anticoagulation should be approached with careful weighing of benefits and risks to identify those who will benefit most from this strategy. This case highlights that even where there is good cardiac function, there is still a major risk of embolic events.

## Declaration of interest

The authors declare that there is no conflict of interest that could be perceived as prejudicing the impartiality of this case report.

## Funding

This work did not receive any specific grant from any funding agency in the public, commercial, or not-for-profit sector.

## Patient consent

Permission has been obtained from the patient’s wife.

## Author contribution statement

S G and R S conceived the manuscript. S G wrote the first draft, which was reviewed by R S.
